# The Role of Cardiovascular Magnetic Resonance in Sports Cardiology; Current Utility and Future Perspectives

**DOI:** 10.1007/s11936-018-0679-y

**Published:** 2018-08-31

**Authors:** Emmanuel Androulakis, Peter P. Swoboda

**Affiliations:** 1 St George’s University Hospitals NHS Foundation Trust, London, UK; 2 Leeds Institute of Cardiovascular and Metabolic Medicine, University of Leeds, Clarendon Way, Leeds, LS2 9JT UK

**Keywords:** Cardiac magnetic resonance imaging, Sports cardiology, Fibrosis, Cardiomyopathies

## Abstract

**Purpose of review:**

Cardiovascular magnetic resonance (CMR) is frequently used in the investigation of suspected cardiac disease in athletes. In this review, we discuss how CMR can be used in athletes with suspected cardiomyopathy with particular reference to volumetric analysis and tissue characterization. We also discuss the finding of non-ischaemic fibrosis in athletes describing its prevalence, distribution and clinical importance.

**Recent findings:**

The strengths of CMR include high spatial resolution, unrestricted imaging planes and lack of ionizing radiation. Regular physical exercise leads to cardiac remodeling that in certain situations can be clinically challenging to differentiate from various cardiomyopathies. Thorough morphological assessment by CMR is fundamental to ensuring accurate diagnosis. Developments in tissue characterization by late gadolinium enhancement and T1 mapping have the potential to be powerful additional tools in this challenging clinical situation. Using late gadolinium enhancement, it is also possible to detect non-ischaemic fibrosis in athletes who do not have overt cardiomyopathy. The mechanisms of this fibrosis are unclear; however, it does appear to be clinically important. We also review data on the prevalence of non-ischaemic fibrosis in athletes.

**Summary:**

CMR is a powerful tool to aid in the diagnosis of cardiomyopathy in athletes. It may also have a future role in assessing fibrosis related to long-term participation in sport.

## Introduction

Long-term exercise leads to structural and functional cardiac adaptation often termed ‘athlete’s heart’. Typical features include left ventricular hypertrophy (LVH), left ventricle (LV) and right ventricle (RV) cavity dilatation and associated electrocardiographic (ECG) changes [1, 2]. Athlete’s heart facilitates an increase in stroke volume and cardiac output as a physiological response to exercise training and may potentially vary across different types of exercise [3].

In clinical practice, it can be challenging to differentiate the physiological changes of athlete’s heart from cardiomyopathy, and in this review article, we will discuss both established and novel cardiovascular magnetic resonance (CMR) techniques that may be helpful for this purpose. Cardiomyopathy is one of the leading causes of death in athletes and often once this diagnosis is made precludes participation in competitive sport. CMR, therefore, has a potentially vital role in improving diagnosis [4].

Recent evidence suggests that prolonged participation in certain sports may predispose certain individuals to cardiac fibrosis, which may be associated with increased risk of arrhythmias, particularly atrial fibrillation [5, 6].

In this systematic review, we discuss the role of CMR in diagnosing cardiomyopathy in athletes and in particular review data on cardiac fibrosis.

## CMR assessment of athlete’s heart

Echocardiography is the first-line investigation to assess cardiac morphology in athletes given its low cost, widespread availability and lack of ionizing radiation. CMR is increasingly used in clinical practice as it allows accurate visualization and quantification of the heart in any plane without limitation by acoustic windows. Using CMR imaging, it is also possible to make an assessment of myocardial tissue characteristics including fat and water content, fibrosis and myocyte mass. By acquiring a stack of short-axis images with no interslice gap with full ventricular coverage, it provides the opportunity to inspect the entire LV myocardium for abnormalities, including focal hypertrophy or regional wall motion abnormalities. Unlike two-dimensional (2D) techniques, cine CMR imaging does not rely on geometric assumptions or calculations based on incomplete sampling of the cardiac volumes [7].

Gadolinium-based CMR contrast agents are exclusively extracellular and can only passively enter damaged cells with a leaky cell membrane. This phenomenon is exploited in late gadolinium enhancement (LGE) CMR imaging. LGE imaging involves administration of typically 0.1–0.2 mmol/kg of a gadolinium-based contrast agent. After a delay of 5–20 min, the contrast agent is retained to a greater extent than in areas of scar or fibrosis than in normal myocardium. The pattern of enhancement on LGE can aid in the diagnosis of cardiomyopathy. For example, subendocardial or transmural LGE is typically seen in ischemic cardiomyopathy, mid wall LGE in dilated and hypertrophic cardiomyopathies and subepicardial LGE in myocarditis [8].

T1 mapping is a method for quantitative assessment of tissue characteristics. The myocardial T1 time can be measured without contrast, native T1, or following the administration of intravenous gadolinium-based contrast agent, post-contrast T1. By combining both these measures, the myocardial extracellular volume (ECV) fraction can be approximated, a value that has been validated histologically in several cardiomyopathies [8].

## Defining athlete’s heart

Morphology of the left ventricle in athlete’s heart has primarily been studied by echocardiography with a clear pattern of LV dilatation and hypertrophy being reported (although varying by sporting type) [1]. The improved reproducibility of CMR has meant that it is possible to validate these findings, with a smaller sample size and in longitudinal studies [9, 10, 11•]. This is exemplified by Arbab-Zadeh et al.[11•] who demonstrated that 1 year of prolonged and intensive endurance training leads to cardiac morphological adaptations in 12 previously sedentary young subjects similar to those observed in elite endurance athletes.

Given the complex structure of the RV morphological assessment by CMR is more reproducible than echocardiography. Increases in RV mass, end diastolic and stroke volumes relative to non-athletes have been described. The ratio of LV to RV size was maintained, leading to the conclusion that athlete’s heart syndrome involves balanced remodeling of both ventricles [10].

## Differentiating physiology from pathology

### Hypertrophic cardiomyopathy

Hypertrophic cardiomyopathy (HCM) is the leading cause of sudden cardiac death in young athletes worldwide [12]. Traditional methods for differentiating physiological left ventricular hypertrophy (athlete’s heart) from HCM have relied on parameters derived from sedentary HCM patients and healthy athletes. As the leading cause of sudden cardiac death in younger populations, the discrimination of HCM from athlete’s heart has immediate clinical relevance [12].

In general, in subjects with good echocardiographic images, CMR provides similar information on ventricular function and morphology but can provide incremental diagnostic value in patients with poor acoustic windows or when some LV regions are poorly visualized— such as the anterolateral wall, the LV apex and the right ventricle [13, 14]. CMR also has a role in identification of subtle markers of HCM including aneurysms, thrombi, myocardial crypts and papillary muscle abnormalities [15].

Cine CMR can be used to differentiate athlete’s heart from pathological LVH using multiple geometric measures. Petersen et al. reported that left ventricular diastolic wall to volume ratio had the highest area under the curve (0.993) providing a sensitivity of 80% and a specificity of 99% to distinguish athlete’s heart from all forms of pathologic LVH [16].

Echocardiography tends to underestimate LA volumes and when assessed by CMR atrial volumes are significantly larger in endurance athletes than controls [17]. The presence of LA dilatation points more towards a diagnosis of athlete’s heart than HCM [18].

HCM is associated with mid wall fibrosis detected on LGE imaging predominantly in areas of hypertrophy and is therefore useful in making the diagnosis. LGE is only present in approximately two thirds of patients with HCM and may be even lower in athletes with HCM [19, 20]. In an athlete with unexplained LV hypertrophy, the presence of typical scar may aid the diagnosis of HCM but its absence cannot exclude it.

**Fig. 1:**
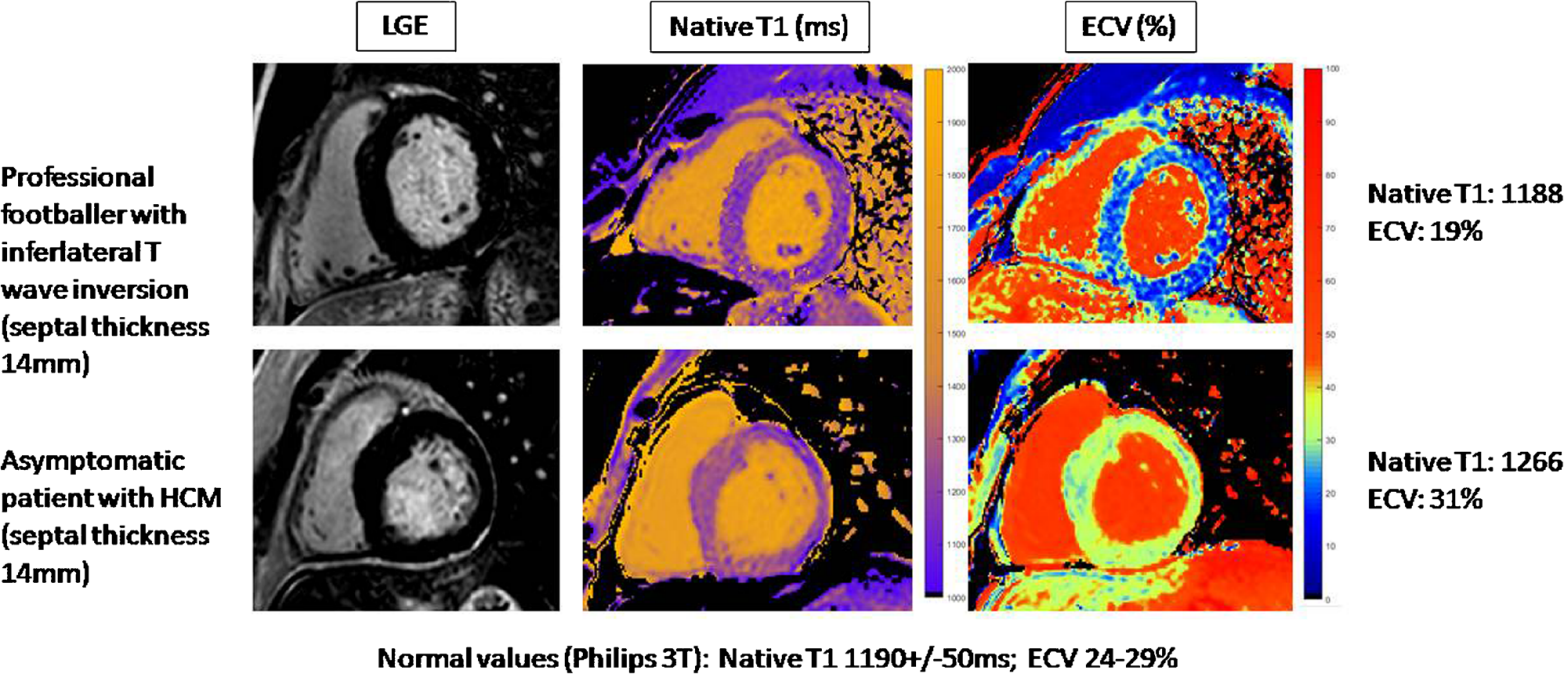
CMR using T1 mapping and ECV has a potential role in the exclusion of HCM in athletes presenting with LV hypertrophy.

Using T1 mapping, it has been shown that in athletes, as fitness increases there is an increase in myocyte mass, with a relatively constant extracellular mass [21]. As shown by Swoboda et al. [22••], the negative correlation between ECV and wall thickness in athletes and sedentary controls suggest that the increase in LV mass in healthy myocardium is mediated by cellular hypertrophy. In HCM, there is a positive correlation between wall thickness suggesting that hypertrophy is mediated by cellular disarray and extracellular matrix expansion. Thus, CMR using T1 mapping has a potential role in the exclusion of HCM in athletes presenting with LV hypertrophy (Fig. 1).

### Arrhythmogenic right ventricular cardiomyopathy

The diagnosis of arrhythmogenic right ventricular cardiomyopathy (ARVC) is based on “Task Force” criteria (TFC) of clinical, histological and electrophysiological and imaging parameters [23]. Imaging criteria require presence of both qualitative findings (RV regional akinesia, dyskinesia, dyssynchronous contraction) and quantitative metrics (decreased ejection fraction or increased indexed RV end-diastolic volume). Major CMR criteria have a sensitivity of 68 to 76%. Minor criteria (RV ejection fraction 40–45% or indexed RV end-diastolic volume 100–110 mL/m^2^ for men and 90–100 mL/m^2^ for women) had a higher sensitivity (79 to 89%), but a consequently lower specificity (85 to 97%) [23]. The distal RV (from the moderator band to the apex) shows highly variable contraction patterns and regional wall motion abnormalities in the sub-tricuspid region are more significant [24]. RV scarring detected on LGE is a common finding in ARVC; however, it is limited by the ability to acquire high-quality LGE images of the RV free wall. This technique is therefore not routinely used [25].

Chronic RV abnormalities and acute dysfunction after endurance sporting events have both been described. Heidbüchel et al. [26] proposed the term “exercise-induced right ventricular cardiomyopathy” in reference to the disproportionate rates of RV dysfunction in elite athletes presenting with serious arrhythmias based on echocardiography, CMR and/or ventriculography. The distinction between ARVC and the effects of exercise are not absolute, and interestingly, exercise may accelerate the development of ARVC in those who are genetically predisposed [27]. On the other hand, a growing body of evidence has suggested that athletes’ RV function is in fact superior to non-athletes as reported by D’Andrea et al. [28]. Therefore, although athletes may have impaired RV function immediately after intense endurance exercise and in the context of ventricular arrhythmias, healthy athletes, at rest, should have RV functional measures that are at least as good as the nonathletic population. La Gerche et al. [29•] tested the hypothesis that intense exercise may promote pro-arrhythmic remodeling in some athletes. Exercise imaging was performed in 17 athletes with RV ventricular arrhythmias, of which eight (47%) had an implantable cardiac defibrillator (ICD), 10 healthy endurance athletes and seven non-athletes. Echocardiographic as well as CMR measures at rest and during intense exercise combined with invasive measurements of pulmonary and systemic artery pressures were obtained. Among athletes with normal cardiac function at rest, exercise testing revealed RV contractile dysfunction among athletes with RV arrhythmias. This is still a matter of ongoing debate that has potentially been underappreciated. With recent advances in RV imaging CMR may potentially elucidate some of these aspects.

### Dilated cardiomyopathy

CMR is the reference technique for the quantification of ventricular volumes and functional parameters, to measure wall thickness and ventricular mass in patients with DCM [30]. Evidence suggests a significantly more heterogeneous end-diastolic LV wall thickness in patients with DCM compared to normal population. Also the physiological gradient in systolic wall thickening between LV basal and apical segments disappears with DCM. RV mass has also been shown to be preserved in DCM patients as compared to normal subjects, whereas LV mass is significantly greater with evidence of larger trabeculae as compared to normal subjects [30, 31], while in advanced cases, LV dysfunction may be associated with diffuse myocardial wall thinning (diastolic wall thickness < 5.5 mm) [30, 31]. LGE has been described as being present in patients with DCM in 12–35% of the cases, the most common pattern being characterized by a midwall linear distribution which might represent the intramural layer of septal fibrosis which has been observed in pathologic samples [30, 32].

In the most extreme athletic training, cardiac remodeling can be profound. It was shown by means of echocardiography on 286 Tour de France cyclists that more than half of the athletes had LV diastolic dimensions exceeding 60 mm and 11.7% had a left ventricular ejection fraction (LVEF) ≤ 52%. This may pose significant difficulty in differentiating athlete’s heart from DCM [33].

If resting measures do not differentiate the athlete from the patients, potentially systolic dysfunction during exercise might be an important aspect. Abernethy et al. [34] demonstrated good augmentation of systolic function in professional footballers with low normal LVEF. Some studies in heart failure populations have observed no exercise induced increment in LVEF using echocardiography whereas others reported some degree of augmentation [32]. Claessen et al. [35] have proposed that contractile reserve can be assessed by exercise CMR and to discriminate physiological and pathological LV remodelling impairment of LV ejection fraction.

Both native T1 and ECV have been shown to be elevated in patients with DCM, even in those without midwall fibrosis [36, 37]. A study comparing early DCM (*N* = 16) and exercisers with low normal LVEF (*N* = 21) showed that exercisers had normal native T1 and ECV whereas both were elevated in those with DCM and this difference could be used to discriminate the two groups [38].

### Left ventricular non-compaction cardiomyopathy

Non-compaction cardiomyopathy occurs due to an autosomally dominant inherited trait in which the middle and apical segments exhibit a thin compact wall with regional dilatation, dysfunction and significant hyper-trabeculation. There are different definitions including an end-diastolic ratio of non-compacted to compacted LV myocardium of greater than or equal to 2.3. Also, LV wall motion abnormalities, global dysfunction or coronary intraventricular thrombi are often present in the disorder [39].

However recently, an unexpectedly large prevalence of LVNCC has been reported in athletes, raising the question of the appropriateness of current diagnostic criteria [40, 41]. In a large athlete population comprising 2501 consecutive athletes who underwent a cardiac evaluation including physical examination, ECG, exercise test and echocardiography and additionally CMR, a marked LV trabecular pattern was seen in 1.4%. Only a small subset of these athletes (0.1%) showed familial, clinical and morphologic changes supporting the diagnosis of LVNCC. It is important to note that the majority of athletes with increased trabeculations were not associated with LV dysfunction and/or positive family history, likely representing a morphologic LV variant, of limited clinical significance [42].

## CMR coronary imaging

Anomalous coronary arteries are relatively common cause of death during sport particularly when the coronaries have a malignant interarterial course [4]. The course of the coronary arteries can be depicted by CMR either with or without administration of contrast [43]. CMR coronary imaging does not expose the athlete to ionizing radiation and is therefore an ideal test for young athletes. Coronary magnetic resonance angiography has been used in a study with 335 individuals (including 207 athletes) with a malignant variant of the right coronary artery identified in four subjects [44].

## Myocardial fibrosis in athletes

Several studies have reported myocardial fibrosis (MF) detected by LGE CMR although the prevalence and pattern have varied (Table 1). However, the causes and mechanisms of MF are unclear. Tahir et al. [47••] comparing 83 asymptomatic triathletes undergoing > 10 training h per week and 36 sedentary controls using LGE and extracellular volume (ECV) CMR demonstrated focal nonischemic MF in 9 of 54 (17%) male triathletes but in none of the female triathletes. Fibrosis was associated with exercise-induced hypertension and the race distances. Merghani et al. [45•] assessed 152 master athletes 54.4 ± 8.5 years of age and 92 matched controls with low Framingham 10-year coronary artery disease risk scores in the aim of identifying the prevalence of subclinical coronary artery disease. Most athletes and controls had a normal coronary artery score (CAC) score; however, male athletes had a higher prevalence of atherosclerotic plaques of any luminal irregularity (44.3 versus 22.2%) compared with sedentary males. Of note, male athletes demonstrated predominantly calcific plaques (72.7%), whereas sedentary males showed predominantly mixed morphology plaques (61.5%) and the number of years of training was the only independent variable associated with increased risk of CAC. Interestingly, in regards to the CMR findings, 14% male athletes but none of the controls revealed LGE, half of whom had a pattern consistent with previous myocardial infarction. Earlier, Wilson et al. [49] having examined 12 lifelong veteran male endurance athletes, 20 age-matched veteran controls and 17 younger male endurance athletes without significant comorbidities, demonstrated that in six (50%) of the veteran athletes, LGE of CMR indicated the presence of myocardial fibrosis and in one case, the pattern was consistent with previous episode of acute myocarditis.

**Table 1:** Recent studies indicating prevalence and patterns of myocardial fibrosis in athletic populations using CMR

Study	Size	Exercise type	Age	Pattern	Prevalence	Associated factors
Merghani [45•]	*n* = 152	Masters endurance athletes	54.4 ± 9 years	7% Ischemic pattern 8% non-ischemic pattern	14% male athletes	No relationship between fibrosis and exercise intensity, years of training, or number of competitions
Breuckmann [46]	*n* = 102	‘Ostensibly’ healthy male runners	61 ± 11 years	5% ischemic pattern 7% non-ischemic pattern	12% prevalence	The event-free survival rate was lower in runners with myocardial LGE than in those without myocardial LGE
Tahir et al.[47••]	*n* = 83	Triathletes	43 ± 10 years	Focal non-ischemic myocardial	17% male athletes	Exercise-induced hypertension and the race distances
Sanchis-Gomar [48]	*n* = 53	11 former ‘elite’ and 42 amateur-level cyclists or runners	55 ± 15 years	Non-ischemic pattern	4% former ‘elite’	No association with any of the biomarkers of fibrosis/remodeling
Wilson [49]	*n* = 12	Competitive endurance veteran athletes	56 ± 6 years	4 veteran athletes with nonspecific cause 1 previous myocarditis 1 silent myocardial infarction	50% of veteran athletes	Number of years spent training, number of competitive marathons and ultra-marathons completed
Schnell [50]	*n* = 7	Asymptomatic athletes recruited during workup of abnormalities on their regular screening examination	26 ± 5 years	Extensive subepicardial LGE predominantly in the lateral wall	100% prevalence as per inclusion criteria	Symptomatic ventricular tachycardia and progressive left ventricular dysfunction

*LGE* late gadolinium enhancement

Also, the amount and intensity of exercise training, as well as the lifelong exercise exposure should be taken into account to test the hypothesis that MF prevalence differs between athletes and the general population.

### Patterns of myocardial fibrosis

There is a growing body of literature showing that the phenotype of MF in athletes demonstrates large variance in patterns, location and quantification. More specifically, MF in athletes has been noticed to be divided into two main categories: non-ischemic and ischemic scar. These phenotypes seem to differ from that in the general population, which may be relevant for the underlying mechanisms and clinical prognosis of MF in apparently healthy athletes. As noted by Merghani et al. [45•], 14% of veteran male athletes but none of the controls revealed LGE, half of whom had a pattern consistent with previous myocardial infarction. On the other hand, Tahir et al. [47••] demonstrated in 83 asymptomatic triathletes using LGE and ECV CMR focal non-ischemic MF in 9 of 54 (17%) males but in none of the female triathletes. In lifelong veteran endurance athletes, MF has been reported near the septal insertion points of the RV free wall in 37% of the athletes with just one subject exhibiting sub-epicardial lateral wall fibrosis [51]. This phenotype seems to differ from that in the general population, which may be relevant for the underlying mechanisms and clinical prognosis of MF in apparently healthy athletes.

### A dose-response relationship?

It is very well established that regular exercise improves cardiovascular risk profile, reduces the risk of myocardial infarction by 50% and is related with a variety of other beneficial effects. Most of these benefits are attributable to moderate exercise. Intense exercise, however, may infrequently trigger arrhythmogenic sudden cardiac death in asymptomatic cardiac disease. Moreover, long-standing vigorous exercise may be associated with adverse electrical and structural remodelling in otherwise normal hearts which may be a dose-response phenomenon [52].

In parallel, evidence from case reports/series suggests that athletes diagnosed as having MF demonstrate high doses of exercise for many years. These studies and case reports suggest a dose-response relationship between lifetime exercise exposure and MF development [53]. Larger studies, such as by Tahir et al. [47••], demonstrated focal non-ischemic MF in 17% males. A cycling race distance of > 1880 km completed during competition had the highest accuracy to predict LGE, with an area under the curve value of 0.876, resulting in high sensitivity (89%) and specificity (79%). Also, the swimming race distance was noted to be independent predictors of LGE presence. La Gerche et al. [54] studied 40 athletes at baseline, immediately following an endurance race (3–11 h duration) and 1-week post-race. LGE localized to the interventricular septum was identified in 5 of 39 athletes who had greater cumulative exercise exposure. Furthermore, in 108 apparently healthy male marathon runners with ≥ 5 marathon competitions during the previous 3 years, the presence of LGE and of coronary artery calcification (CAC) in relation to cardiovascular risk factors was evaluated. It was noted that CAC percentile values and number of marathons independently predicted the presence of LGE [55].

In contrast, another report provided evidence of potential myocardial injury and ventricular dysfunction after prolonged exercise using CMR imaging before and after amateur marathon races in 28 healthy males. Although marathon running led to a transient increase of cardiac biomarkers, no detectable myocardial necrosis was observed as evidenced by LGE [56]. It is important to note that existing data is conflicting and the majority lacks direct verification of functional myocardial alterations by CMR especially in large number of athletes. Larger, well-designed studies, based on well-defined criteria of athletes’ populations are needed in order to reach safe conclusions.

### Clinical implications

The presence of MF is an important risk factor for adverse cardiac outcomes in clinical populations. However, the prognostic value of MF has not been extensively studied in athletic population [51]. The impact of lifelong exercise training is still a matter of ongoing debate. Although there are data supporting a U-shaped association between exercise volume and cardiovascular risk, most available evidence suggests a curvilinear relationship, with greater health benefits at larger exercise doses [51, 57]. Several studies have identified the presence of LGE in the heart of extensively trained veteran athletes. La Gerche et al. [47••] have shown myocardial fibrosis by CMR and a reduction in RV systolic function in athletes with long-term exercise, suggesting that the heart has a limited capacity to tolerate the overload exercise. Some authors have also a new entity, the so called “Phidippides cardiomyopathy”; long-term strenuous exercise can induce cardiac dilation and also activates resident macrophages, pericytes and fibroblasts, resulting in the deposition of collagen and fibrosis. It may be possible that the RV is more susceptible to fatigue than the left ventricle after prolonged exercise although more studies are required to identify a probable effect of exercise “dose” and their implication in the development of heart failure [58].

In addition, nonischemic LV scar, which is characteristically localized at the mid-myocardial or subepicardial layers of the LV wall, can be found as mentioned above, in a broad spectrum of heart muscle diseases at risk of sudden cardiac death (SCD), including myocarditis, sarcoidosis, DCM, HCM and ARVC. Whether this isolated, segmental LV myocardial lesion may act as a substrate of life-threatening arrhythmias and SCD in the athlete remains to be elucidated. Also, the presence of LGE at the junction of the RV with the ventricular septum has been consistently reported in a sizeable proportion of endurance athletes and related to the duration and intensity of sports activity but this pattern is traditionally deemed a non-clinically relevant; however, follow-up studies evaluating its arrhythmic risk are lacking [53]. Zorzi et al. [59] evaluated 35 athletes with ventricular arrhythmias by CMR and compared with 40 healthy control athletes. A stria LGE pattern with subepicardial/midmyocardial distribution, mostly involving the lateral LV wall, was found in 27 (77%) versus 0 in controls, whereas a spotty pattern of LGE localized at the junction of the right ventricle to the septum was, respectively, observed in 11 (31%) versus 10 (25%) though this did not reach statistical significance.

It is important to emphasize that the prognostic significance of nonspecific MF patterns seen in athletes is yet to be clarified as there is no significant evidence currently, that athletes with this pattern should be restricted from exercise.

## Conclusions

Multimodality cardiac imaging has been used in all of its forms to study the intrigues of the athlete’s heart syndrome. A description of the changes in cardiac morphology and function has permitted this syndrome to be differentiated from serious cardiac pathology, which may mimic it in many cases. Cardiac imaging has not successfully and conclusively answered this question, although recent advances in CMR who great promise. MF has been reported in endurance athletes and the pattern of LGE is heterogeneous, which may represent different causes and prognostic significance. Larger, well-designed studies, based on well-defined criteria of athletes’ populations are needed in order to reach safe conclusions.

## Abbreviations

CMR: Cardiovascular magnetic resonance
ECV: extracellular volume
LV: left ventricle
